# Comparison the Incidence and Severity of Side Effects Profile Of FOLFOX and DCF Regimens in Gastric Cancer Patients

**DOI:** 10.22037/ijpr.2019.1100663

**Published:** 2019

**Authors:** Ebrahim Salehifar, Razieh Avan, Ghasem Janbabaei, Seyed Khalil Mousavi, Fatemeh Faramarzi

**Affiliations:** a *Board certified Clinical Pharmacist, Pharmaceutical Research Center, Mazandaran University of Medical Sciences, Sari, Iran. *; b *Board Certified Pharmacotherapy Specialist, Assistant Professor of Clinical Pharmacy, Medical Toxicology and Drug Abuse Research Center (MTDRC), Faculty of Medicine, Birjand University of Medical Sciences, Birjand, Iran. *; c *Board certified Hematologist Oncologist, Gastrointestinal Research Center, Mazandaran University of Medical Sciences, Sari, Iran. *; d *Student Research Committee, Faculty of Pharmacy, Mazandaran University of Medical Sciences, Sari, Iran.*

**Keywords:** Gastric cancer, Chemotherapy, Adverse reactions, FOLFOX, DCF

## Abstract

Gastric cancer is the fourth common cancer and the second leading cause of cancer death worldwide. Due to lack of adequate information on the side effects of chemotherapy regimens in treatment of gastric cancer, this study was aimed to determine the side effects of two common chemotherapy regimens of gastric cancer. This prospective study was conducted in Emam Khomeini Educational Hospital and Touba Polyclinic; both are affiliated to Mazandaran University of Medical Sciences. The frequency and severity of side effects of chemotherapy were recorded based on the National Cancer Institution (NCI) Toxicity Criteria (version 2). DCF (Docetaxel, Cisplatin, 5FU) and FOLFOX (Folinic acid, 5FU, Oxaliplatin) adverse reactions were compared using SPSS 16 software. One hundred twenty five chemotherapy cycles administered to seventy four patients were assessed. The most common used regimens were DCF (70%) and FOLFOX (16%). The incidence of vomiting was higher with DCF compared to FOLFOX (P = 0.049). In more than 50% of cycles, DCF regimen caused diarrhea, while in FOLFOX regimen it was less than 9% (P = 0.002). Stomatitis, visual changes, nausea, skin reactions, and constipation were not significantly different between the two regimens. It seems that the adverse drug reactions of FOLFOX regimen were more favorable than DCF regimen. The results of this study may help clinicians choosing a more favorable chemotherapy regimen especially in patients with a low performance status who have difficulties in tolerating a chemotherapy regimen with a more severe adverse effect profile.

## Introduction

Gastric cancer is the fourth common cancer and the second leading cause of cancer death worldwide, though the incidence has been dwindling for several decades. Gastric cancer is among the common cancers in Asia, Latin America, Central and Eastern Europe (1). In Iran, considering both genders, gastric cancer is the most frequent cause of cancer death (2). Although incidence and mortality due to gastric cancer has been decreased in most countries such as United States, the actual number of new patients is rising annually which is mainly due to the population aging. The incidence of gastric cancer has been widely growing in Iran, especially in Northern regions such as Mazandaran province (3). The National Comprehensive Cancer Network (NCCN) Clinical Practice Guidelines in Oncology for Gastric Cancer recommends a multidisciplinary approach for treatment of gastric cancer patients (4). Cancer treatment is usually a combination of different treatment modalities including surgery, radiotherapy, and chemotherapy. Choosing an appropriate treatment depends on the stage of the disease, being prepared for surgery, preference by the patient, the overall situation of the disease, and also its comorbidities (3). In initial stages, the main priority is removing the lesion by surgery. According to the applied investigations, this approach leads to improvement of survival of the patients. At second and third stages, chemotherapy and radiotherapy are applied as neoadjuvant or adjuvant therapy along with surgery which is helpful in improvement of the survival of the patients (5). The main regimens in neoadjuvant chemotherapy are FOLFOX (Folinic acid, 5FU, Oxaliplatin), DCF (Docetaxel, Cisplatin, 5FU), ECF (Epirubicin, Cisplatin, 5FU), ECF- modified, and EOX (Epirubicin, Oxaliplatin) regimens (6). The efficacy and safety of each chemotherapy regimen should be considered together to have a favorable cancer management. One study compared the efficacy and safety of two regimens DCF and ECF. It has been observed that both regimens were generally associated with grade 1-2 side effects and showed similar toxicity profiles (7). Another study compared the DCF regimen to other non-taxane-containing palliative chemotherapy and indicated that DCF regimen had a tolerable toxicity profile with a better response rate than other non-taxane-containing chemotherapy, though the febrile neutropenia was a little more common with DCF regimen (8). A study pointed out the efficacy and toxicity of chemotherapy regimens used in advanced stages of gastric cancer. FOLFOX, DCF and FOLFIRI were among the most frequent regimens. Grades 3-4 mucositis was found in 30.8% of patients treated with DCF regimen. Grades 3-4 leukopenia was occurred in 42.3%, 8.3% and 36.4% of patients received DCF, FOLFOX and FOLFIRI regimens, respectively, though the toxicity profile, as well as the efficacy of these regimens, was not statistically different (9). Analysis of efficacy and safety of the mFOLFOX-6 and DCF regimens demonstrated that these two regimens are not different in terms of hematological toxicities. The incidence of grade 3-4 nausea/vomiting and diarrhea was significantly more common with DCF regimen (10).

As far as we know, this is the first study compares the safety of two main regimens in treatment of gastric cancer in Iran. Regarding lack of our knowledge about the side effects profile of chemotherapy regimens used in our patients, this research was conducted to explore the incidence and severity of side effects caused by common chemotherapy regimens used in treatment of gastric cancer patients in north of Iran. 

## Experimental

This prospective study was conducted on the patients with gastric cancer diagnosis who were been treated in the Emam Khomeini educational hospital or Touba polyclinic, both are affiliated to Mazandaran University of Medical Sciences in 2013-2014. The proposal of the study was approved by the Research Council of Mazandaran University of Medical Sciences. Patients studied and signed the informed consent form before enrollment. All gastric cancer patients who received chemotherapy were eligible to enter the study. The examined side effects in this study include nausea, vomiting, diarrhea, constipation, hair loss, stomatitis, neuropathy, skin reactions and changes in vision. The severity of side effects has been classified according to the National Cancer Institution (NCI) Toxicity Criteria (version 2). Statistical analysis has been done with SPSS version 16. Descriptive statistics have been used to describe the incidence and severity of side effects of chemotherapy regimens, and also the Chi-square test has been used to compare qualitative data for the two groups. The sum of percentage of grade 0 and 1 (e.g., no toxicity or mild toxicity) was compared with the sum of grade 2 and 3 (moderate to high toxicity) to recognize the more favorable regimen. P < 0.05 was considered as statistically significant difference.

## Results

In this study, One hundred forty five chemotherapy cycles administered to seventy four patients were prospectively assessed in terms of adverse events. The most common chemotherapy regimens were DCF (70%) and FOLFOX (16%), respectively. Demographic and clinical data of patients and the type of chemotherapy regimens used for patients have been presented in Table 1.

**Table 1:** Demographic and clinical data of patients

Sex	
Male	50 (67.6%)
Female	24 (32.4%)
Age (year)	
Mean	61.9
SD	14.3
Min	26
Max	91
Median	63
Stage (%)	
1	0 (0%)
2	2 (6.1%)
3	5 (15.2%)
4	26 (78.8%)
Site of Tumor	
Cardia	12 (35.3%)
Body	5 (14.7%)
Antrum	11(32.4%)
Lesser curvature	6 (17.6%)
Greater curvature	0 (0%)
Fundus	0 (0%)
Type of Tumor	
Adenocarcinoma	44 (100%)
Other types	0 (0%)
Chemotherapy Regimens	
DCF	102 (70%)
FOLFOX	23 (16%)
ECF	6 (4.1%)
EOF	5 (3.5%)
XELOX	3 (2.1%)
CF	3 (2.1%)
DOF	2 (1.4%)
PCF	1 (0.7%)

CF: Cisplatin, 5FU; DCF: Docetaxel, Cisplatin, 5FU; DOF: Docetaxel, Oxaliplatin, 5FU; DOX: Docetaxel, Oxaliplatin, Xeloda; ECF: Epirubicin, Cisplatin, 5FU; EOX: Epirubicin, Oxaliplatin; FOLFOX: Folinic acid, 5FU, Oxaliplatin; PCF: Paclitaxel, Cisplatin, 5FU; XELOX: Xeloda, Oxaliplatin

**Table 2:** Outcome variables of different adverse effects of chemotherapy regimens

**Adverse Reactions (%)**	**FOLFOX** **Grade of Adverse Reactions**				**DCF** **Grade of Adverse Reactions**				***P*** **-value** *
	**0**	**1**	**2**	**3**	**0**	**1**	**2**	**3**	
Nausea	26.1	13	60.9	0	13.7	16.7	65.7	3.9	0.41
Vomiting	26.1	0	43.5	30.4	19.6	3.9	65.7	10.8	0.049
Skin reactions	100	0	0	0	90.2	3.9	4.9	1	0.48
Diarrhea	91.3	0	8.7	0	48	9.8	36.3	5.9	0.002
Constipation	73.9	4.3	21.7	0	89.2	1	9.8	0	0.13
Stomatitis	82.6	13	4.3	0	88.2	6.9	4.9	0	0.61
Visual Problems	87	0	0	13	90.2	2.9	1	5.9	0.48
Hair loss	91.3	8.7	0	0	32.4	28.4	39.2	0	<0.001

* P-value: Chi-square test for comparing the grades of adverse effects. The sum of percentage of grade 0 and 1 (e.g., no toxicity or mild toxicity) was compared with the sum of grade 2 and 3 (moderate to high toxicity) to recognize the more favorable regimen.

Due to the limited number of patients in most regimens, only the data related to the DCF (102 cycles) and FOLFOX (23 cycles) regimens were used to compare the side effects. The incidence and severity of DCF and FOLFOX regimens side effects were shown in Table 2 according to NCI criteria.

Incidence and severity of nausea were not significantly different between DCF and FOLFOX regimens (P = 0.41). The severity of most experienced nausea was Grade 2 (65.7% and 60.9% for DCF and FOLFOX, respectively). The incidence of vomiting was higher with DCF regimen compared to FOLFOX (79.4% vs. 73.9%) (P = 0.049). With DCF regimens, most patients had Grade 2 vomiting (65.7 % of all the patients). However, the percentage of patients who had grade 3 vomiting was higher in FOLFOX regimen (30.4% vs. 10.8%). Skin reactions were observed in 8 % of DCF cycles, while none of the FOLFOX cycles was associated with skin problems. Statistically, the difference between the two groups was not significant (P = 0.48). In more than 50% of cycles, DCF caused diarrhea, while in FOLFOX regimen it was less than 9%. DCF regimen was associated with a more frequent and more severe diarrhea (P = 0.002). Visual disturbances have occurred in 9.8% and 13% of DCF and FOLFOX cycles, respectively. Differences in the two groups were not significant (P = 0.48). In the case of neuropathy, the difference between the two regimens was significant (P = 0.022). Neuropathy was occurred approximately in half of the DCF cycles, while the rate of neuropathy with FOLFOX cycles was 17.3%. In terms of hair loss, there are considerable differences between the two groups (P < 0.001). Hair loss was occurred in 68% of DCF cycles and only in 9% of FOLFOX cycles. No patients in the FOLFOX group experienced total alopecia, whereas approximately 40 percent of patients in DCF cycles experienced total alopecia. Maximum severity of the stomatitis was grade 2, which observed in less than 5% of DCF and FOLFOX cycles. Stomatitis with FOLFOX regimen was somewhat more common (17.3% in FOLFOX group vs. 12% in DCF group). Constipation was recorded in more than 25% of FOLFOX and 11% of DCF cycles. No significant difference in the incidence of constipation was observed between DCF and FOLFOX cycles (P = 0.13).

## Discussion

To the best our knowledge, this study is the first research that compares the safety of two popular chemotherapy regimens in patients with gastric cancer in Iran. This study was performed to evaluate and compare the side effects of chemotherapy regimes in gastric cancer patients. There is currently no single standard regimen as first-line treatment of gastric cancer. Most chemotherapy regimens consist of two or three drugs and are based on cisplatin and fluoropyrimidines (11). NCCN guidelines suggest DCF regimen as a first-line treatment of advanced gastric cancer (12). Van Cutsem *et al* reported that DCF regimen versus CF regimen were associated with stomatitis in 59% and 60% of cases, diarrhea in 75% and 46% of cases, nausea in 72% and 75% of cases, vomiting in 61% and 71% of cases and sensory neuropathy in 38% and 24% of cases, respectively (13). Our study indicates more incidence of nausea and vomiting and fewer diarrhea and stomatitis in patients who received DCF regimen, compare to this study. In contrast, in terms of the severity of side effects, less grades 3-4 gastrointestinal toxicity (e.g., nausea, vomiting, diarrhea and stomatitis) was occurred in our study. Another study also compared the adverse reactions of DCF and CF regimens. Grade 3-4 diarrhea was more common with DCF regimen (20% vs. 8%) and grade 3-4 stomatitis were less common with DCF (21% vs. 27%) (14). Our study has shown less grade 3-4 diarrhea and stomatitis compared to this study. Ajani has compared the efficacy and safety of DCF regimen against CF regimen in patients with gastric cancer. Grade 3-4 of mucositis was more common with CF (21% vs. 27%) and diarrhea was more common with DCF (19% vs. 8%) (15). Our study demonstrated less incidence of grade 3-4 diarrhea and mucositis in recipients of DCF regimen. 

Atarian *et al* studied the chemotherapy regimens for advanced unresectable gastric cancer. 

The study was conducted on 56 patients who totally received 274 cycles of DCF, gastrointestinal toxicity occurred in 50% of patients including mucositis in 20% and diarrhea in 16% of patients. In addition, 10% of patients experienced neuropathy (16). Our study indicates more incidence of diarrhea and neuropathy and less mucositis with DCF regimen. Teker *et al* have compared the adverse reaction profile of DCF and ECF regimens. Four hundred five cycles of chemotherapy (48% DCF and 52% ECF regimens) were studied. Comparing DCF vs. ECF, Nausea/vomiting, diarrhea, stomatitis was reported in 52.4% vs. 50%, 0% vs. 4.5% and 0% vs. 6.8% of patients, respectively (7). Our study has reported more nausea/vomiting, diarrhea, and stomatitis than the study of Teker *et al*. In a systematic and meta-analysis review, DCF regimen was compared to non-taxane-containing palliative chemotherapy. The incidence of diarrhea, nausea/vomiting, stomatitis, constipation and alopecia with DCF was 58.9%, 59.2%, 56.2%, 26.3%, and 73.5%, respectively (8). In terms of the incidence of gastrointestinal toxicity, the frequency of all side effects, except nausea/vomiting, was less with DCF regimen in our study. Since 2001, the FOLFOX regimen has been introduced as one of the most effective treatments for advanced gastric cancer (17). Several studies have shown the efficacy and tolerability of the oxaliplatin, 5FU, and leucovorin (FOLFOX-4, modified FOLFOX-4, FOLFOX-6, and modified FOLFOX-6) in patients with metastatic gastric cancer. Louvet *et al.* have conducted the phase II study of FOLFOX-6 regimen on patients with advanced or metastatic gastric cancer. Grade 3-4 nausea, vomiting, diarrhea, stomatitis, alopecia and grade 3 peripheral neuropathy (severe) were observed in 6%, 0%, 4%, 9%, 0%, and 21% of the patients, respectively (18). Luo *et al*. have conducted the pilot study of FOLFOX-6 regimen on patients with advanced or recurrent gastric cancer. Grade 3-4 nausea, vomiting, diarrhea, stomatitis, alopecia and sensory neuropathy have been observed at 0%, 9.8%, 5.9%, 0%, 3.9%, and 5.9% of the patients, respectively (19). De Vita *et al*. evaluated the toxicity and clinical efficacy of FOLFOX-4 regimen in patients with advanced gastric cancer. Grade 3 nausea, vomiting and diarrhea were observed at 5%, 2% and 5% of the patients, respectively. Grade 3 peripheral neuropathy was also reported in 5% of the patients (20). In our study, with FOLFOX regimen, all the mentioned side effects were less in comparison with three recent studies except vomiting. In South Korea, Kim *et al* have compared the DCF, FOLFOX, and FOLFIRI regimens. Among 1203 patients, 568 patients received chemotherapy regimens (around 47%). Totally 51 patients (9%) had a complete response to treatment, which 12 of them were on FOLFOX regimen, 11 of them were on FOLFIRI regimen and 26 patients on DCF regimen. Grade 3-4 mucositis was observed in 30.8% of patients treated with DCF regimen. The incidence of nausea/vomiting with FOLFOX, DCF, and FOLFIRI regimens were reported 58.3%, 80.8%, and 54.6%, respectively. Moreover, the incidence of diarrhea in FOLFOX, DCF, and FOLFIRI regimens were 0%, 7.6%, and 9.1%, respectively (9). Hacibekiroglu *et al.* conducted analysis of the efficacy and safety of two mFOLFOX-6 and DCF regimens and showed that hematologic toxicity between two regimens are not different. The incidence of nausea/vomiting, diarrhea and peripheral neuropathy with FOLFOX and DCF regimens were 7.4% vs. 20.8%, 5.6% vs. 19.4%, and 5.6% vs. 4.2%, respectively. The incidence of grade 3-4 nausea/vomiting and diarrhea with DCF regimen was higher compared to FOLFOX regimen (10). In our study, DCF regimen rather than FOLFOX regimen showed greater nausea, vomiting, diarrhea and neuropathy as like as two recent studies. By comparative analysis of two DCF and FOLFOX regimen in this prospective study, the severity of neuropathy, vomiting, hair loss, and diarrhea were significantly higher with DCF regimen which is similar to above studies. According to the results of various studies and this research, non-hematologic side effects of DCF regimen were more frequent compared to FOLFOX.

Currently, there is no standard chemotherapy regimen in the treatment of advanced gastric cancer (21).

Although the DCF regimen frequently has been used to treat patients with advanced stage of gastric cancer, high level of toxicity (grade 3-4) with this regimen has been reported (13, 20, 22).

In several studies, the FOLFOX regimen is the most common used chemotherapy regimen for advanced gastric cancer with effectiveness and low level of toxicity (23, 24, 25). As mentioned above, only two studies evaluated the efficacy and safety of DCF and FOLFOX regimens for treatment of advanced gastric cancer. In Korean population, the efficacy and safety of two DCF and FOLFOX regimens were not significantly different in treatment of advanced gastric cancer (9). In another study, there was no statistically significant difference between the DCF and mFOLFOX-6 regimens with regard to efficacy, but the non-hematological toxicities of DCF regimen was more than mFOLFOX-6 regimen (10). Our findings are in agreement with recent two studies that compare the safety of DCF and mFOLFOX-6 regimens in advanced gastric cancer.

According to our results, it seems that FOLFOX regimen may be the optimal regimen especially for patients with low performance status who cannot tolerate adverse effects of chemotherapy (e.g., old patients). 

The low number of patients especially in FOLFOX regimen, as well as other chemotherapy regimens used in gastric cancer, is one of the limitations of our study. Due to lack of enough cases of other chemotherapy regimens, a through comparison between all of gastric cancer chemotherapy regimens were not possible. However, the main purpose of the study was to compare the two most commonly used chemotherapy regimens, FOLFOX and DCF, in gastric cancer patients. 

## Conclusion

Based on the results of this study, gastrointestinal toxicity (vomiting and diarrhea), hair loss, and neuropathy are more common with DCF regimen rather than FOLFOX regimen. Stomatitis, visual disturbances, nausea, skin reactions, and constipation were not significantly different in those two regimens. It seems that the toxicity profile of FOLFOX regimen was more favorable than DCF regimen.

## References

[1] Ferro A, Peleteiro B, Malvezzi M, Bosetti C, Bertuccio P, Levi F, Negri E, Vecchia CL, Lunet N (2014). Worldwide trends in gastric cancer mortality (1980–2011), with predictions to 2015, and incidence by subtype. Eur. J. Cancer.

[2] Movahedi M, Afsharfard A, Moradi A, Moadeli AN, Khoshnevis J, Fattahi F, Akbari ME (2009). Survival rate of gastric cancer in Iran. J. res. med. sci.

[3] Mousavi SK, Janbabai G, Kouchaki B, Borhani H, Rashidi M, Salehifar E (2015). Demographic and clinical characteristics of gastric cancer patients in north of Iran, Mazandaran province, 2008-2014. Pharm. Biomed. Res.

[4] Ajani JA, Bentrem DJ, Besh S, D′Amico TA, Das P, Denlinger C, Fakih MG, Fuchs CS, Gerdes H, Glasgow RE, Hayman JA, Hofstetter WL, Ilson DH, Keswani RN, Kleinberg LR, Michael Korn W, Craig Lockhart A, Meredith K, Mulcahy MF, Orringer MB, Posey JA, Sasson AR, Scott WJ, Strong VE, Varghese Jr TK, Warren G, Kay Washington M, Willett C, Wright CD, McMillian NR, Sundar H (2013). Gastric cancer, version 22013: featured updates to the NCCN Guidelines. J Natl Compr Canc Netw.

[5] Cunningham D, Allum WH, Stenning SP, Thompson JN, Van de Velde CJ, Nicolson M, Scarffe JH, Lofts FJ, Falk SJ, Iveson TJ, Smith DB, Langley RE, Verma M, Weeden S, Chua YJ (2006). Perioperative chemotherapy versus surgery alone for resectable gastroesophageal cancer. N Engl J Med.

[6] Chen W, Shen J, Pan T, Hu W, Jiang Z, Yuan X, Wang L (2014). FOLFOX versus EOX as a neoadjuvant chemotherapy regimen for patients with advanced gastric cancer. Exp Ther Med.

[7] Teker F, Yilmaz B, Kemal Y, Kut E, Yucel I (2014). Efficacy and safety of docetaxel or epirubicin, combined with cisplatin and fluorouracil (DCF and ECF), regimens as first line chemotherapy for advanced gastric cancer: a retrospective analysis from Turkey. Asian Pac J Cancer Prev.

[8] Chen XL, Chen XZ, Yang C, Liao YB, Li H, Wang L, Yang K, Li K, Hu JK, Zhang B, Chen ZX, Chen JP, Zhou ZG (2013). Docetaxel, cisplatin and fluorouracil (DCF) regimen compared with non-taxane-containing palliative chemotherapy for gastric carcinoma: a systematic review and meta-analysis. PloS one.

[9] Kim YJ, Goh PG, Kim ES, Lee SY, Moon HS, Lee ES, Sung JK, Kim SH, Lee BS, Jeong HY (2011). Comparison of the toxicities and efficacies of the combination chemotherapy regimens in advanced gastric cancer patients who achieved complete response after chemotherapy. Korean J Gastroenterol.

[10] Hacibekiroglu I, Kodaz H, Erdogan B, Turkmen E, Esenkaya A, Onal Y, Uzunoglu S, Cicin I (2015). Comparative analysis of the efficacy and safety of modified FOLFOX-6 and DCF regimens as first-line treatment in advanced gastric cancer. Mol Clin Oncol.

[11] Ochenduszko S, Puskulluoglu M, Konopka K, Fijorek K, Urbanczyk K, Budzynski A, Matlok M, Lazar A, Sinczak-Kuta A, Pedziwiatr M, Krzemieniecki K (2015). Comparison of efficacy and safety of first-line palliative chemotherapy with EOX and mDCF regimens in patients with locally advanced inoperable or metastatic HER2-negative gastric or gastroesophageal junction adenocarcinoma: a randomized phase 3 trial. Med Oncol.

[12] Dong L, Li J, Lou XP, Miao JH, Lu P, Chang ZW, Han ZF (2014). Comparison of short-term efficacy and safety of TIROX and DCF regimens for advanced gastric cancer. J Int Med Res.

[13] Van Cutsem E, Moiseyenko VM, Tjulandin S, Majlis A, Constenla M, Boni C, Rodrigues A, Fodor M, Chao Y, Voznyi E, Risse ML, Ajani JA (2006). Phase III study of docetaxel and cisplatin plus fluorouracil compared with cisplatin and fluorouracil as first-line therapy for advanced gastric cancer: a report of the V325 Study Group. J. Clin. Oncol.

[14] Moiseyenko VM, Ajani J, Tjulandin S, Majlis A, Constenla M, Boni C, Anelli A, Yver AJ, Van Cutsem E (2005). Final results of a randomized controlled phase III trial (TAX 325) comparing docetaxel (T) combined with cisplatin (C) and 5-fluorouracil (F) to CF in patients (pts) with metastatic gastric adenocarcinoma (MGC). J. Clin. Oncol.

[15] Ajani JA (2006). The role of docetaxel in gastric cancer. EJC Suppl.

[16] Atarian H, Ghadyani M, Rezvani H, Abasahl AG (2007). The effective chemotherapy regimen for advanced unresectable gastric cancer. Iranian Journal of Surgery.

[17] Sun Z, Zhu RJ, Yang GF, Li Y (2014). Neoadjuvant chemotherapy with FOLFOX4 regimen to treat advanced gastric cancer improves survival without increasing adverse events: a retrospective cohort study from a Chinese center. Scientific World J.

[18] Louvet C, Andre T, Tigaud JM, Gamelin E, Douillard JY, Brunet R, Franc¸ois E, Jacob JH, Levoir D, Taamma A, Rougier P, Cvitkovic E, de Gramont A (2002). Phase II study of oxaliplatin, fluorouracil, and folinic acid in locally advanced or metastatic gastric cancer patients. J. Clin. Oncol.

[19] Luo HY, Xu RH, Zhang L, Li YH, Shi YX, Lin TY, Han B, Wang F, Qiu MZ, He YJ, Guan ZZ (2008). A pilot study of oxaliplatin, fluorouracil and folinic acid (FOLFOX-6) as first-line chemotherapy in advanced or recurrent gastric cancer. Chemotherapy.

[20] De Vita F, Orditura M, Matano E, Bianco R, Carlomagno C, Infusino S, Damiano V, Simeone E, Diadema MR, Lieto E, Castellano P, Pepe S, De Placido S, Galizia G, Di Martino N, Ciardiello F, Catalano G, Bianco AR (2005). A phase II study of beweekly oxaliplatin plus infusional 5fluorouracil and folinic acid (FOLFOX4) as first-line treatment of advanced gastric cancer patients. Br. J. Cancer, BJC.

[21] Bernards N, Creemers GJ, Nieuwenhuijzen GA, Bosscha K, Pruijt JF, Lemmens VE (2013). No improvement in median survival for patients with metastatic gastric cancer despite increased use of chemotherapy. Ann Oncol.

[22] Roth AD, Fazio N, Stupp R, Falk S, Bernhard J, Saletti P, Koberle D, Borner MM, Rufibach K, Maibach R, Wernli M, Leslie M, Glynne-Jones R, Widmer L, Seymour M, de Braud F (2007). Docetaxel, cisplatin, and fluorouracil; docetaxel and cisplatin; and epirubicin, cisplatin, and fluorouracil as systemic treatment for advanced gastric carcinoma: a randomized phase II trial of the Swiss Group for Clinical Cancer Research. J Clin Oncol.

[23] Wang LB, Shen JG, Xu CY, Chen WJ, Song XY, Yuan XM (2008). Neoadjuvant chemotherapy versus surgery alone for locally advanced gastric cancer: a retrospective comparative study. Hepatogastroenterology.

[24] Zhang J, Chen RX, Cai J, Meng H, Wu GC, Zhang ZT, Wang Y, Wang KL (2012). Efficacy and safety of neoadjuvant chemotherapy with modified FOLFOX7 regimen on the treatment of advanced gastric cancer. Chinese medical journal.

[25] Moon H, JF J WA, Liu YH, Zhang ZT, Wang S (2007). Oxaliplatin plus 5-fuorouracil/leueovorin (FOLFOX7) as neoadjuvant plus adjuvant treatment versus adjuvant alone in locally advanced resectable gastric cancer: BJSA-01 study design and interim results. Gastrointestinal Cancers Symposium, Abstract.

